# Translation of Questionnaires Measuring Health Related Quality of Life Is Not Standardized: A Literature Based Research Study

**DOI:** 10.1371/journal.pone.0127050

**Published:** 2015-05-12

**Authors:** Anne Kjaergaard Danielsen, Hans-Christian Pommergaard, Jakob Burcharth, Eva Angenete, Jacob Rosenberg

**Affiliations:** 1 Department of Nursing, Faculty of Health and Technology, Metropolitan University College, Copenhagen, Denmark; 2 Department of Surgery, Herlev Hospital, University of Copenhagen, Herlev, Denmark; 3 Department of Surgery, Institute of Clinical Sciences, Sahlgrenska Academy at the University of Gothenburg, SSORG, Scandinavian Surgical Outcomes Research Group, Sahlgrenska University Hospital/Östra, Gothenburg, Sweden; University of Nebraska Medical Center, UNITED STATES

## Abstract

**Introduction:**

There is growing awareness of the need to explore patient reported outcomes in clinical trials. In the Scandinavian Surgical Outcomes Research Group we are conducting several clinical trials in cooperation between Danish and Swedish surgical researchers, and we use questionnaires aimed at patients from both countries. In relation to this and similar international cooperation, the validity and reliability of translated questionnaires are central aspects.

**Main Objectives:**

The purpose of this study was to explore which methodological measures were used in studies reporting translation of questionnaires. Furthermore, we wanted to make some methodological suggestions for clinical researchers who are faced with having to translate a questionnaire.

**Material and Methods:**

We designed a research study based on a survey of the literature and extracted data from published studies reporting the methodological process when translating questionnaires on health related quality of life for different diseases.

**Results:**

We retrieved 187 studies and out of theses we included 52 studies. The psychometric properties of the translated versions were validated using different tests. The focus was on internal validity (96%), reliability (67%) criterion validity (81%), and construct validity (62%). For internal validity Cronbach's alpha was used in 94% of the studies.

**Conclusions:**

This study shows that there seems to be a consensus regarding the translation process (especially for internal validity) although most researchers did not use a translation guide. Moreover, we recommended that clinical researchers should consider three steps covering the process of translation, the qualitative validation as well as the quantitative validation.

## Introduction

In recent years there has been growing interest in incorporating patient reported outcomes in clinical research [1]. Health-related quality-of-life is a central patient reported outcome, and has become more accepted as a valid goal within patient treatment [2]. Often researchers want to use the same survey instrument in more than one country, and an important example would be Short Form—36 (SF-36) [3] that has been translated into several languages across continents.

The translation process must be able to cover all steps needed to ensure that the validity and reliability of the questionnaire is still intact when translating a questionnaire originally developed in another country and culture [4]. However, when an original questionnaire has once been validated in the original version, one might argue that it is context specific and that the translated questionnaire would not be an equivalent survey instrument. The validation of the translated questionnaire covers a qualitative and a quantitative part, including aspects that cover understanding of human expression in written language. Evidence of an appropriate translation methodology is lacking and different translation methods have not been fully evaluated [5].

When including patient reported outcome using questionnaires, the instrument must be valid, reliable, and if measuring change over time, also responsive [6]. The validity of a questionnaire might be broken down into face validity and content validity, where reliability focuses on the reproducibility of the measurements [7]. Responsiveness is related to construct validity, and the instrument should be able to differentiate between observed differences or changes [8]. With the growing number of studies conducted as international multicenter studies, the need for cultural adaptation of the original questionnaire is evident [9].

A literature review suggested that translation of questionnaires should be guided by a check list [10] in order to ensure high cultural as well as linguistic quality. However, the check list only focused on the translation process, and did not cover the validation of the translation. This may be because translation and validation are separate methodological processes, although one is logically followed by the other during the translational process, and as such appear connected. The Patient Reported Outcomes Measurement Information System (PROMIS) does provide researchers, clinicians and patients with different resources, however, not as a single and easily accessible document [11]. Translation includes the process of transforming the questionnaire, whereas the validation process primarily covers the process of quality assessment of the translated tool.

In the Scandinavian Surgical Outcomes Research Group (SSORG) [12] we are conducting several clinical trials in cooperation between Danish and Swedish surgical researchers. We use questionnaires dealing with health related quality of life in Danish and Swedish patients who we include in our studies [13,14]. Therefore, we often discuss the validity and reliability of the questionnaires that we apply in our studies.

The overarching aims of this study were two-fold: firstly we wanted to explore what methodological measures were used in studies reporting translation of questionnaires. Secondly, we hoped to identify specific methodological interventions that could guide the clinical researcher, not only when translating a questionnaire but also when validating the process. More specifically, we wanted to evaluate how often a guideline or checklist was used and whether this had any consequences for the choice of method of translation and validation. Our focus was on the different methods used by the researchers to validate the translational process. In this sense, we were interested in both the qualitative as well as the quantitative validation processes.

## Material and Methods

We designed a research study based on a survey of the literature, and aimed at extracting data from published studies reporting the methodological process when translating questionnaires. We used in part the methodology of a systematic review, as described in Preferred Reporting Items for Systematic Reviews and Meta-Analyses (PRISMA) [15], but we did not do any systematic assessment of the validity of the retrieved studies, either related to the external or the internal dimensions of validity [16]. We assumed that focusing on linguistic and cultural aspects as well as the psychometric properties would make it possible for us to illuminate the issues sufficiently.

We did a systematic and comprehensive database search [15], and searched Cinahl, Medline, Embase, and Cochrane. We used the following search terms for Medline: Questionnaires [MeSH Terms], quality of life [MeSH Terms], translation [Title/Abstract], validation [Title/Abstract], "validation studies"[Publication Type], filters: humans, English. Subsequently, the search string was adapted to the other databases. All hits were screened according to our research question (PICO)[17]: **P**: Questionnaires exploring health related quality of life, **I:** Validation of language translation of the questionnaire, **C**: N/A, **O:** Methods describing and/or testing the language translation process.

Eligibility criteria were: (1.) studies translating questionnaires aimed at health related quality of life (hrqol), irrespective of whether this would be generic or disease-specific, (2.) translations from English to a European language (S1 Fig).

Four researchers (AKD, HCP, JB, EA) conducted the final inclusion of studies, and the data extraction. Data extraction was preceded by a standardization process including reflections on the methodological issues, and construction of a checklist for inclusion of studies and extraction of data. The checklist was tested by all four researchers together using 10 of the included studies, and then it was adjusted accordingly. The remaining studies were divided between the four researchers for data extraction. One author (AKP) did a final check of the data extraction of the included studies, and any inconsistencies were settled by all authors.

We extracted the following data in each study: Study ID, type of questionnaire, assessment area, translation guide, forward/backward translation, professional translator, expert panel review, mother tongue same as target language, target audience, lay persons. The method of validation was noted regarding internal validity, criterion validity, and construct validity.

### Statistical analysis

All data were analysed using SPSS IBM 20 (IBM Corp. Released 2011. IBM SPSS Statistics for Windows, Version 20.0. Armonk, NY: IBM Corp) and Microsoft Office Excel 2007. Data were analysed using descriptive and non-parametric tests, reporting median (range), mean (SD), and percentage where applicable. Differences between groups were analysed using Chi-square and Fisher´s exact test and a *p*-value of less than 0.05 was considered statistically significant.

### Ethical approval

The study was exempted from approval by the Danish Ethical Committee as well as the Danish Data Protection Agency as we did not include any form of biomedical intervention or any personal data related to individual and identifiable humans.

## Results

**Fig 1:**
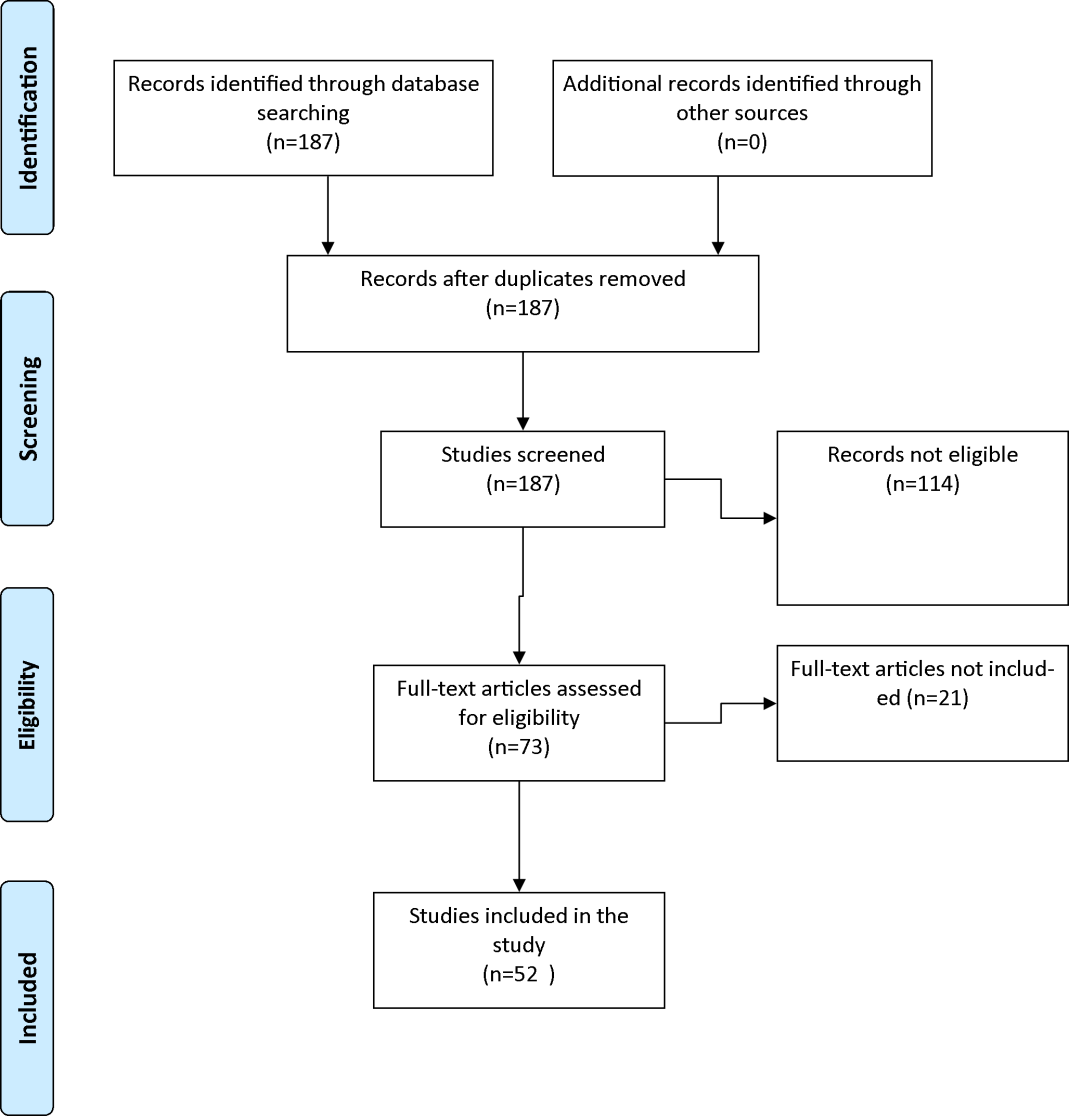
PRISMA 2009 Flow Diagram.

Among the 187 studies screened, 52 studies were selected (Table 1). Reasons for papers not meeting inclusion criteria were: no information regarding the translational process, no health related quality of life questionnaires, or they did not report translation from English to a European language (Fig 1). Of the included studies, two (3.8%) studies translated questionnaires aimed at generic health related quality of life, and 50 (96.2%) translated disease specific questionnaires. The total sum of participants in all the included studies was 7761.

**Table 1:** Showing assessment area for each study included in the analysis.

Group ID	Assessment area	Study Id number
1	Sclerosis	1,7,20,25,38,45
2	Asthma	2,14,33
3	Muscle/joint	3,12,16,19,21,22,24,48,52
4	Psychiatry	4
5	Obesity	5,8
6	Fecal incontinence	6
7	Dermatology	9,11,28,44,46,51
8	Heart disease	10,15,17
9	Dentistry	13,42,43
10	Nephrology	18,30,37
11	Cancer	23,37
12	Growth hormone	39,41
13	Oto-rhino-laryngology	26,31,50,
14	Allergy	27
15	Dementia	29,36
16	Epilepsy	32
17	Cystic fibrosis	34
18	Dyspepsia	40
19	Gynaecology	47
20	IBD	49

Most studies did not use a translation guide (n 36, 83%), but both forward and backward translation were commonly used (n 41, 79%). Almost half of the studies used a professional translator (n 25, 48%), and most used a translator with the same mother tongue as the target audience (n 38, 73%).

More than half of the studies (n 26, 50%) included a qualitative validation using an interview with the target audience; and 13 of the included studies (25%) used the method of face-to-face validation. Whether lay persons were used in the validation process was not reported in 41 of the studies (79%).

The psychometric properties of the translated versions were generally validated using different tests,.. Focus was on internal validity (n 50, 96%), reliability (n 35, 67%), criterion validity (n 42, 81%), and construct validity (n 32, 62%). For internal validity Cronbach's alpha was used in 94% of the studies (n 49, n 60), and reliability was in 17 cases tested using Intra Class Correlation (30.8%) (Table 2 and S2 Fig).

**Table 2:** Presenting numbers of variables (showing percentages in brackets) related to the phases of translation, the qualitative validation and the quantitative validation.

Phases	Variable	Number (percentage) of studies reporting the method
**Translation**	Translation guide	16(31)
	Forward/backward translation	41(79)
	Professional translators	25(48)
	Translators with mother tongue the same as the target audience	38(73)
	Review by expert panel	39(75)
**Qualitative validation**	Interview of target audience	26(50)
	Testing of face-validity	13(25)
	Lay persons	11(21)
**Quantitative validation**	Internal validity	50(96)
	Tested with Cronbach´s alpha	49(94)
	Reliability	35(67)
	Tested with Intra Class Correlation	17(33)
	Criterion validity	42(81)
	Construct validity	32(62)

We could not find any statistical evidence for the assumption that if researchers had access to a translation guideline they would be impelled to apply forward, forward/backward translation (Fisher’s exact test, *p* = *0*.*5*), professional translators (Fisher’s exact test, *p* = 1), review by expert panels (Fisher’s exact test, *p* = *0*.*8*), or use of lay persons (Fisher’s exact test, *p* = *0*.*7*). Neither did it affect the validation process, internal validity (Chi-square *p* = *0*.*5*), reliability (Chi-square, *p* = 0.7), criterion validity (Fisher’s exact test, *p* = 0.7), or construct validity (Fisher’s exact test, *p* = *1*).

## Discussion

This study pointed at some consensus regarding the translation process although most researchers did not use a translation guide. The consensus was especially centred on the use of forward/backwards-translations and use of Cronbach´s alpha when testing the internal validity. Our hypothesis, that a guideline or checklist would affect which tests and methods that would be used, was not supported, as we did not detect any significant differences regarding this question. This is interesting as many experts in the field strongly recommend this [5,9,10,18,19, 20]. Yet the recommendations, guidelines and checklists did not seem to cover both the translation as well as testing of the translation, which might mask the fact that clinical researchers possibly need checklists that focus on the entire process. As such, the results of our study may reflect the actual use of guidelines and checklists, altogether missing either the translation or the testing.

The studies reviewed in this article focused on the linguistic and semantic translational process of the forward/backward translation. The backward translation has been recommended in a set of standardized guidelines [9], but has also been declared counterproductive by other researchers claiming that the backward translation does not assess the quality of the translation [21,22]. To resolve this, a multistep approach during translation with registration and documentation of each phase has been suggested [10] as well as the use of a two-panel approach employing both professional and lay translators in the process [23]. In this study, most included studies reported using backward translation. However, the use of a multistep approach was not described and thus not explored in our study, and therefore the reconciliation of the different phases of forward and backward translation was not displayed, and could not be analysed further.

Although there are recommendations to use professional translators [24], our results suggest that researchers often replace this by review in expert panels. In this way, both the linguistic and the clinical expertise would be included in the validation process. Another recommendation that more than half of the studies had followed was to include the target population for testing the translation [9]. Involvement of the target population may be done in different ways, and we found that interviewing was the preferred method in our sample. However, in the literature there are recommendations of face-validity alone [7] or in combination with use of the probe technique as pre-testing [9,18], which was not reported or commented on in the studies included. Moreover, guidelines recommended inclusion of lay persons, which was rarely reflected or used in the included studies [21].

When turning to the psychometric properties of the translated questionnaire, several questions have to be answered. The internal validity was often assessed in the included studies, predominantly using Cronbach´s alpha. Cronbach´s alpha should be computed for each subscale separately as it intends to calculate the principal concept by drawing on multiple items [25]. A Cronbach´s alpha measures the correlation between subscales and is generally advised to be between 0.70 and 0.90, as a measure of good internal consistency; however, it has previously been argued that the use of Cronbach’s alpha as the only measure is insufficient [26].

Conversely, the reliability was only tested in 67% of the studies and with the use of Intra Class Correlations (ICC) test in 33% of the studies. Using ICC would explore the degree of reproducibility in repeated measurements [7] and would assess consistency between multiple observers in the same sample [27]. Our results pointed at 33% not reporting this issue at all, and 33% not being specific about the method.

However, the construction of questionnaires was often guided by the classical test theory (CTT), which makes certain assumptions about the items and the scale statistics. First; that the scale is dependent on the specific sample of persons who were the target group, secondly; the question of item equivalence here CTT is assumed to have equivalent variances [7]. These assumptions are connected to the direct relationship between the actual items used in the questionnaire and the construct underlying it. Newer theories suggest using models addressing latent variables such as item response theory (IRT) and structural equation models (SEM) that seem to be able to overcome the limitations of CTT [28].

The translation of questionnaires needs to address the linkage between the measurement tool and the persons being measured, the implications of not applying other tests might alter the original questionnaire dramatically. For instance, IRT is applied in order to focus more on the influence of the individual and the attribute being measured [29]. On the other hand, SEM is useful to identify the connection between latent variables, as IRT (as well as CTT) is based on one-dimensional scales as opposed to SEM being based on the assumption of a relationship on several dimensions. The two tests have received increasing attention, but as they were not manifest in our study and we did not pursue the analytical differences in detail. This should probably be incorporated in future studies developing scales and questionnaires or translating questionnaires.

Assessment of criterion validity should give answers related to concurrent and predictive validation. The concurrent validation of the questionnaire may be tested in parallel with a gold standard, and the predictive validation may be treated as a diagnostic test [7]. More than 80% of the studies explored concurrent validity by testing the correlation with another scale. The construct validity was only tested in a little more than half of the included studies. It evaluates the consistency with the theoretical constructs of the scale being measured [25], and perhaps it is sufficient that it is tested during the construction of the original questionnaire.

The limitations of this study were probably the design of the study, as we did a research study based on a literature search and hereby relying on the quality of the search method. This meant that studies may not have been included if the indexation differed from the one our search covered. Furthermore, this study was descriptive and therefore any comparisons between different validation methods and techniques were not examined. However, as the aim of the study was to explore the methods reported by clinical researchers, we believe that the methods were valid and sound. Furthermore, the study did not explore specific details related to different analyses testing the psychometric properties of the included questionnaires, as we believe that this information was beyond the scope of this study.

## Conclusions

Studies translating questionnaires measuring health related quality of life showed some agreement related to the process of translation (forward/backward translation) and the following testing of psychometric properties with qualitative methods (interview with target group, or face-validity testing) and quantitative methods (internal validity and reliability). We were able to point to several methodological actions that should be considered when translating questionnaires addressing health related quality of life.

Item 1 translational process: Forward/backward translation, including either a professional translator, a translator with the same tongue as target audience, an expert review group, or a lay-person translator.

Item 2 qualitative validation: Including either interview with target audience, or face-to-face validation.

Item 3 quantitative validation: Including one or more tests focusing on internal validity, reliability, criterion validity, and/or construct validity.

These actions could be seen as methodological steps that may be used and subsequently reported when translating questionnaires.

The suggested three steps should be considered when clinical researchers embark on into translating questionnaires aimed at health related quality of life.

## Supporting Information

S1 Fig Abstract screening sheet. (XLSX)

S2 Fig Showing full references for all included studies. (DOCX)
