# Supplementary material for: Translation of Questionnaires Measuring Health Related Quality of Life Is Not Standardized: A Literature Based Research Study
Source: PLoS One. 2015 May 12;10(5):e0127050. doi: 10.1371/journal.pone.0127050 (PMC4428794; doi:10.1371/journal.pone.0127050)
Supplement: S2 Fig — (DOCX) [file pone.0127050.s002.docx]

**S2 Supporting information file: Showing full references for all included studies.**

| Number | Full reference | Assess-ment area | Trans-lation guide | Forward/forward-backward | Trans-lation guide | Expert panel review | Experts mother tongue as target | Use of target audience | Internal validity | Relia-  bility | Criterion validity | construct validity |
| --- | --- | --- | --- | --- | --- | --- | --- | --- | --- | --- | --- | --- |
| 1 | Füvesi J, Bencsik K, Benedek K, Mátyás K, et al. Cross-cultural adaptation and validation of the “Multiple Sclerosis Quality of Life Instrument” in Hungarian. Mult Scler 2008;14:391–8. | 1 | - | FW* | + | + | + | - | CA*** | - | - | + |
| 2 | Ferreira J, Silveira P, Figueiredo MM, Andrade C, et al. Validation of the Portuguese version of the Asthma Quality of Life Questionnaire [AQLQ-M] by Marks. Rev Port Pneumol 11:351–66 | 2 | - | FW/BW** | + | + | + | face validity | CA | - | - | + |
| 3 | Gonzalez Sáenz de Tejada M, Escobar A, Herdman M, Herrera C, et al. Adaptation and validation of the Osteoarthritis Knee and Hip Quality of Life (OAKHQOL) questionnaire for use in patients with osteoarthritis in Spain. Clin Rheumatol 2011;30:1563–75 | 3 | - | FW/BW | + | - | - | interview | CA | ICC**** | + | + |
| 4 | Berle JØ, McKenna SP. Quality of Life in Depression Scale (QLDS): adaptation and evaluation of the psychometric properties of the Norwegian version. Nord J Psychiatry 2004;58:439–46 | 4 | - | FW | - | - | - | interview | CA | other | + | + |
| 5 | Charalampakis V, Daskalakis M, Bertsias G, Papadakis JA, et al. Validation of the Greek translation of the obesity-specific Moorehead-Ardelt Quality-of-Life Questionnaire II. Obes Surg 2012;22:690–6. | 5 | - | FW/BW | + | + | + | - | CA | ICC | + | - |
| 6 | Dehli T, Martinussen M, Mevik K, Stordahl A, et al. Translation and validation of the Norwegian version of the fecal incontinence quality-of-life scale. Scand J Surg 2011;100:190–5 | 6 | - | FW/BW | + | - | - | face validity | CA | ICC | + | - |
| 7 | Ciudin A, Franco A, Diaconu MG, Peri L, et al. Quality of life of multiple sclerosis patients: translation and validation of the Spanish version of Qualiveen. Neurourol Urodyn 2012;31:517–20 | 1 | - | FW/BW | + | - | - | interview | CA | other | + | - |
| 8 | Bilbao A, Mar J, Mar B, Arrospide A, et al. Validation of the Spanish translation of the questionnaire for the obesity-related problems scale. Obes Surg 2009;19:1393–400 | 5 | - | FW/BW | - | + | + | interview | CA | - | + | + |
| 9 | Augustin M, Wenninger K, Amon U, Schroth MJ, et al. German adaptation of the Skindex-29 questionnaire on quality of life in dermatology: validation and clinical results. Dermatology 2004;209:14–20 | 7 | - | FW/BW | + | + | + | - | CA | - | + | + |
| 10 | De Gucht V, Van Elderen T, van der Kamp L, Oldridge N. Quality of life after myocardial infarction: translation and validation of the MacNew Questionnaire for a Dutch population. Qual Life Res 2004;13:1483–8 | 8 | - | FW/BW | - | + | + | - | CA | - | + | + |
| 11 | Valero A, Herdman M, Bartra J, Ferrer M, et al. Adaptation and validation of the Spanish version of the Chronic Urticaria Quality of Life Questionnaire (CU-Q2oL). J Investig Allergol Clin Immunol 2008;18:426–32 | 7 | - | FW/BW | + | + | + | interview | CA | - | + | + |
| 12 | Ornetti P, Parratte S, Gossec L, Tavernier C, et al. Cross-cultural adaptation and validation of the French version of the Knee injury and Osteoarthritis Outcome Score (KOOS) in knee osteoarthritis patients. Osteoarthritis Cartilage 2008;16:423–8 | 3 | - | FW/BW | - | + | + | interview | CA | ICC | + | - |
| 13 | Östberg AL, Andersson PIA, Hakeberg M. Cross-cultural adaptation and validation of the oral impacts on daily performances (OIDP) in Swedish. Swed Dent J 2008;32:187–95 | 9 | - | FW/BW | + | + | + | interview | - | ICC | - | + |
| 14 | Somerville A, Knöpfli B, Rutishauser C. Health-related quality of life in Swiss adolescents with asthma: Validation of the AAQOL-D and comparison with Australian adolescents. Swiss Med Wkly 2004;134:91–6 | 2 | - | FW/BW | - | - | - | interview | CA | - | + | + |
| 15 | Somerville A, Knöpfli B, Rutishauser C. Health-related quality of life in Swiss adolescents with asthma: Validation of the AAQOL-D and comparison with Australian adolescents. Swiss Med Wkly 2004;134:91–6 | 8 | + | FW | - | + | + | interview | - | - | - | - |
| 16 | Opara J, Szary S, Kucharz E. Polish cultural adaptation of the Roland-Morris Questionnaire for evaluation of quality of life in patients with low back pain. Spine (Phila Pa 1976) 2006;31:2744–6 | 3 | - | FW/BW | - | + | + | - | CA | - | + | - |
| 17 | Scarinzi C, Berchialla P, Ghidina M, Rozbowsky P, et al. The Severe Heart Failure Questionnaire: Italian translation and linguistic validation. J Prev Med Hyg 2008;49:152–7 | 8 | - | FW/BW | + | + | + | - | CA | - | + | + |
| 18 | Yildirim A, Ogutmen B, Bektas G, Isci E, et al. Translation, Cultural Adaptation, Initial Reliability, and Validation of the Kidney Disease and Quality of Life-Short Form (KDQOL-SF 1.3) in Turkey. Transplant Proc 2007;39:51–4 | 10 | - | FW/BW | + | + | + | - | CA | other | + | - |
| 19 | Viehoff PB, van Genderen FR, Wittink H. Upper limb lymphedema 27 (ULL27): Dutch translation and validation of an illness-specific health-related quality of life questionnaire for patients with upper limb lymphedema. Lymphology 2008;41:131–8 | 3 | - | FW/BW | - | - | - | interview | CA | - | + | - |
| 20 | Pekmezovic T, Kisic Tepavcevic D, Kostic J, Drulovic J. Validation and cross-cultural adaptation of the disease-specific questionnaire MSQOL-54 in Serbian multiple sclerosis patients sample. Qual Life Res 2007;16:1383–7 | 1 | + | FW/BW | + | + | + | interview | CA | - | - | + |
| 21 | Verspaget M, Nicolaï SPA, Kruidenier LM, Welten RJTJ, et al. Validation of the Dutch version of the Walking Impairment Questionnaire. Eur J Vasc Endovasc Surg 2009;37:56–61. | 3 | - | FW/BW | - | + | + | - | CA | ICC | + | + |
| 22 | Teleman P, Stenzelius K, Iorizzo L, Jakobsson U. Validation of the Swedish short forms of the Pelvic Floor Impact Questionnaire (PFIQ-7), Pelvic Floor Distress Inventory (PFDI-20) and Pelvic Organ Prolapse/Urinary Incontinence Sexual Questionnaire (PISQ-12). Acta Obstet Gynecol Scand 2011;90:483–7 | 3 | + | FW | - | + | + | - | CA | ICC | + | - |
| 23 | Zotti P, Del Bianco P, Serpentini S, Trevisanut P, et al. Validity and reliability of the MSKCC Bowel Function instrument in a sample of Italian rectal cancer patients. Eur J Surg Oncol 2011;37:589–96 | 11 | - | FW/BW | - | - | - | interview | CA | ICC | + | + |
| 24 | Huber W, Hofstaetter JG, Hanslik-Schnabel B, Posch M, et al. The German version of the Oxford shoulder score Cross-cultural adaptation and validation. Arch Orthop Trauma Surg 2004;124:531–6 | 3 | - | FW/BW | + | + | + | - | CA | other | - | - |
| 25 | Katsarou Z, Bostantjopoulou S, Peto V, Alevriadou A, et al. Quality of life in Parkinson’s disease: Greek translation and validation of the Parkinson's disease questionnaire (PDQ-39). Qual Life Res 2001;10:159–63 | 1 | - | FW/BW | - | - | + | interview | CA | other | - | + |
| 26 | Khaldoun E, Woisard V, Verin É. Validation in French of the SWAL-QOL scale in patients with oropharyngeal dysphagia. Gastroenterol Clin Biol 2009;33:167–71 | 13 | - | FW | - | - | - | face validity | CA | - | - | - |
| 27 | Kiotseridis H, Cilio CM, Bjermer L, Aurivillius M, et al. Swedish translation and validation of the Pediatric Allergic Disease Quality of Life Questionnaire (PADQLQ). Acta Paediatr Int J Paediatr 2011;100:242–7 | 14 | + | FW/BW | - | + | + | face validity | CA | ICC | + | + |
| 28 | Litleré Moi A, Wentzel-Larsen T, Salemark L, Hanestad B. Validation of a Norwegian version of the Burn Specific Health Scale. Burns 2003;29:563–70 | 7 | + | FW/BW | + | + | + | interview | CA | ICC | + | + |
| 29 | Lucas-Carrasco R, Gómez-Benito J, Rejas J, Brod M. The Spanish version of the dementia quality of life questionnaire: a validation study. Aging Ment Health 2011;15:482–9 | 15 | - | FW/BW | - | + | + | - | CA | ICC | + | + |
| 30 | Malindretos P, Sarafidis P, Spaia S, Sioulis A, et al. Adaptation and Validation of the Kidney Disease Quality of Life-Short Form Questionnaire in the Greek Language. Am J Nephrol 2009;9–14 | 10 | - | FW | - | + | + | interview | CA | other | - | + |
| 31 | Marro M, Mondina M, Stoll D, de Gabory L. French validation of the NOSE and RhinoQOL questionnaires in the management of nasal obstruction. Otolaryngol Head Neck Surg 2011;144:988–93 | 13 | - | FW | - | - | - | face validity | CA | other | + | + |
| 32 | Martinović Ž, Milovanović M, Tošković O, Jovanović M, et al. Psychometric evaluation of the Serbian version of the Quality of Life in Epilepsy Inventory-31 (QOLIE-31). Seizure 2010;19:517–24 | 16 | - | FW | + | + | + | interview | CA | other | + | + |
| 33 | Miedinger D, Chhajed PN, Stolz D, Leimenstoll B, et al. Reliability and validity of a German asthma quality of life questionnaire. Swiss Med Wkly 2006;136:89–95 | 2 | + | FW/BW | - | + | + | - | CA | ICC | + | + |
| 34 | Monti F, Lupi F, Gobbi F, Agostini F, et al. Validation of the Italian version of the Cystic Fibrosis Quality of Life Questionnaire (CFQoL), a disease specific measure for adults and adolescents with cystic fibrosis. J Cyst Fibros 2008;7:116–22 | 17 | + | FW/BW | + | + | + | face validity | CA | other | + | + |
| 35 | Nazar G, Garmendia ML, Royer M, McDowell JA, et al. Spanish validation of the University of Washington Quality of Life questionnaire for head and neck cancer patients. Otolaryngol Head Neck Surg 2010;143:801–7, 807.e1–2 | 11 | + | FW/BW | + | + | - | face validity | CA | ICC | + | - |
| 36 | Novelli MMPC, Dal Rovere HH, Nitrini R, Caramelli P. Cross-cultural adaptation of the quality of life assessment scale on Alzheimer disease. Arq Neuropsiquiatr 2005;63:201–6 | 15 | 0 | FW/BW | - | + | + | face validity | CA | other | + | - |
| 37 | Øksnes M, Bensing S, Hulting AL, Kam̈pe O, et al. Quality of life in European patients with Addison’s disease: Validity of the disease-specific questionnaire AddiQoL. J Clin Endocrinol Metab 2012;97:568–76. | 10 | 0 | FW/BW | + | + | + | - | other method | other | + | + |
| 38 | Jamroz-Wisniewska A, Stelmasiak Z, Bartosik-Psujek H. Validation analysis of the Polish version of the Multiple Sclerosis International Quality of Life Questionnaire (MusiQoL). Neurol Neurochir Pol 2011;45:235–44. | 1 | + | FW/BW | + | - | - | interview | CA | other | + | + |
| 39 | Karbownik-Lewińska M, Lewiński A, McKenna S, Kokoszko A, et al. The Polish version of the Quality of Life Assessment of Growth Hormone Deficiency in Adults (QoL-AGHDA) - Four-stage translation and validation. Endokrynol Pol 2008;59:374–84 | 12 | + | FW | - | + | + | face validity | CA | other | + | + |
| 40 | Kulich KR, Reguła J, Stasiewicz J, Jasinski B, et al. Psychometric validation of the Polish translation of the Gastrointestinal Symptom Rating Scale (GSRS) and Quality of Life in Reflux and Dyspepsia (QOLRAD) Questionnaire in patients with reflux disease. Pol Arch Med Wewn 2005;113:241–9. | 18 | + | FW/BW | - | - | - | face validity | CA | other | + | + |
| 41 | McKenna SP, Wilburn J, Twiss J, Crawford SR, et al. Adaptation of the QoL-AGHDA scale for adults with growth hormone deficiency in four Slavic languages. Health Qual Life Outcomes 2011;9:60 | 12 | + | FW/BW | - | + | + | interview | CA | other | + | + |
| 42 | Hassel AJ, Rolko C, Koke U, Leisen J, et al. A German version of the GOHAI. Community Dent Oral Epidemiol 2008;36:34–42. | 9 | 0 | FW/BW | + | + | + | face validity | CA | ICC | - | - |
| 43 | Cortés-Martinicorena FJ, Rosel-Gallardo E, Artázcoz-Osés J, Bravo M, et al. Adaptation and validation for Spain of the child-oral impact on daily performance (C-OIDP) for use with adolescents. Med Oral Patol Oral Cir Bucal 2010;15. | 9 | 0 | FW/BW | + | + | + | interview | CA | ICC | + | - |
| 44 | Dominguez AR, Balkrishnan R, Ellzey AR, Pandya AG. Melasma in Latina patients: Cross-cultural adaptation and validation of a quality-of-life questionnaire in Spanish language. J Am Acad Dermatol 2006;55:59–66 | 7 | 0 | FW/BW | - | + | + | interview | CA | - | + | - |
| 45 | DAncona CAL, Tamanini JT, Botega N, Lavoura N, et al. Quality of life of neurogenic patients: Translation and validation of the Portuguese version of Qualiveen. Int Urol Nephrol 2009;41:29–33 | 1 | + | FW/BW | - | + | + | face validity | CA | ICC | + | - |
| 46 | Dreno B, Finlay AY, Nocera T, Verrière F, et al. The cardiff acne disability index: Cultural and linguistic validation in French. Dermatology 2004;208:104–8 | 7 | + | FW/BW | + | + | + | interview | CA | ICC | + | - |
| 47 | Giannarini G, Keeley FX, Valent F, Milesi C, et al. The Italian Linguistic Validation of the Ureteral Stent Symptoms Questionnaire. J Urol 2008;180:624–8 | 19 | 0 | FW/BW | + | + | + | interview | CA | other | + | - |
| 48 | Hedin P-J, McKenna SP, Meads DM. The Rheumatoid Arthritis Quality of Life (RAQoL) for Sweden: adaptation and validation. Scand J Rheumatol 35:117–23 | 3 | 0 | FW | - | + | + | interview | CA | other | + | + |
| 49 | Ciccocioppo R, Klersy C, Russo ML, Valli M, et al. Validation of the Italian translation of the Inflammatory Bowel Disease Questionnaire. Dig Liver Dis 2011;43:535–41 | 20 | 0 | FW/BW | - | + | + | face validity | CA | other | + | + |
| 50 | Andersson O, Ryden A, Ruth M, Ylitalo Moller R, et al. Validation of the Swedish translation of LPR-HRQL. Med Sci Monit 2010;16:CR480–R487. | 13 | 0 | FW/BW | - | + | - | interview | CA | - | + | + |
| 51 | Chmielowska K, Tomaszewski KA, Pogrzebielski A, Brandberg Y, et al. Translation and validation of the Polish version of the EORTC QLQ-OPT30 module for the assessment of health-related quality of life in patients with uveal melanoma. Eur J Cancer Care (Engl) 2013;22:88–96. | 7 | + | FW | + | - | + | interview | CA | - | + | + |
| 52 | Schuster C, McCaskey M, Ettlin T. German translation, cross-cultural adaptation and validation of the whiplash disability questionnaire. Health Qual Life Outcomes 2013;11:45 | 3 | + | FW/BW | + | + | - | interview | CA | - | + | - |

* Forward translation only ** Forward/backward translation *** Cronbach´s alpha **** Intra Class Correlation
